# 1523. Association between Self-reported Masking Behavior and SARS-CoV-2 Infection Wanes from Pre-Delta to Omicron-Predominant Periods — North Carolina COVID-19 Community Research Partnership (NC-CCRP)

**DOI:** 10.1093/ofid/ofac492.085

**Published:** 2022-12-15

**Authors:** Ashley H Tjaden, Michael A Gibbs, Michael S Runyon, William Weintraub, Yhenneko Taylor, Sharon Edelstein

**Affiliations:** George Washington University, Rockville, MD; Atrium Health - Carolinas Medical Center, Charlotte, North Carolina; Atrium Health Department of Emergency Medicine, Charlotte, North Carolina; MedStar Health Research Institute and Georgetown University, Washington, District of Columbia; Atrium Health, Charlotte, North Carolina; George Washington Univ Biostatistics Center, Rockville, Maryland

## Abstract

**Background:**

Wearing a face mask is a primary public health method to reduce SARS-CoV-2 transmission. We assessed the association between self-reported mask use and risk of COVID-19 infection during three periods of the pandemic.

**Methods:**

We performed a nested case-control analysis within the NC-CCRP of adults ≥18 years who completed daily syndromic surveillance surveys from April 2020 through February 2022, comparing self-reported cases to controls who never self-reported a positive test. We matched up to 10 controls to each case on calendar time of self-reported positive test and corresponding daily survey entry. Not wearing a mask was defined as responding “no” at least once in the ten days preceding the match date to “In the last 24 hours, have you worn a face mask or face covering every time you interacted with others (not in your household) within a distance of less than 6 feet?” Conditional logistic regression models of risk of COVID-19 infection were adjusted for demographics, vaccination status, and recent known exposure to COVID-19. We tested any days not wearing a mask during the Pre-Delta (July 1 2020-June 30, 2021), Delta (July 1– November 30, 2021), and Omicron (December 1, 2021 – February 28, 2022) periods.

**Results:**

Among 3,901 cases and 27,813 date-matched controls, there was a significant interaction between mask use and time period (p< 0.001). Prior to July 2021, the odds of a reported SARS-CoV-2 infection was 66% higher (aOR=1.66, 95% CI=1.43 – 1.91) among participants reporting at least one day not wearing a mask compared to those who reported no days (1592 cases, 11717 controls). During the Delta-predominant period, the results were similar (aOR=1.53, 95% CI=1.23 – 1.89; 659 cases, 4649 controls). This association was attenuated during the Omicron-predominant period, where the odds of a reported SARS-CoV-2 infection was 16% higher (aOR=1.16, 95% CI=1.03 – 1.32; 1563 cases, 10960 controls).

**Conclusion:**

While the effect of not wearing a mask remains significant, during the Omicron-predominant period we observed a decrease in the association between self-reported mask wearing and risk of SARS-CoV-2 infection. The increased transmissibility of Omicron, pandemic fatigue, and increasing population immunity are possible contributing factors.

**Disclosures:**

**Michael S. Runyon, MD, MPH**, Abbott Laboratories: Grant/Research Support|Roche Diagnostics Operations, Inc: Grant/Research Support.

